# Short- and long-term prognosis of acute critically ill patients with systemic rheumatic diseases: A retrospective multicentre study

**DOI:** 10.1097/MD.0000000000026164

**Published:** 2021-09-03

**Authors:** Paul Chabert, William Danjou, Mehdi Mezidi, Julien Berthiller, Audrey Bestion, Abla-Akpene Fred, Claude Guerin, Laurent Argaud, Vincent Piriou, Eric Bonnefoy-Cudraz, Jean-Jacques Lehot, Jean-Luc Fellahi, Thomas Rimmele, Frederic Aubrun, Jean-Christophe Richard, Laure Gallay, Arnaud Hot

**Affiliations:** a Hospices Civils de Lyon, Médecine Intensive - Réanimation, Hôpital de la Croix Rousse, Lyon, France; b Hospices Civils de Lyon, Pôle Santé-Publique, Lyon, France; c Hospices Civils de Lyon, Département Information Médicale, France; d Université de Lyon, France; e Hospices Civils de Lyon, Médecine Intensive - Réanimation, Hôpital Edouard Herriot, Lyon, France; f Institut Mondor de Recherche Biomédicale INSERM 955, ERL CNRS, 7000 Créteil, France; g Hospices Civils de Lyon, Anesthésie-Réanimation-Médecine Intensive, Centre Hospitalier Lyon Sud, Pierre-Bénite, France; h Hospices Civils de Lyon, Service d’urgences cardiologiques et de soins intensifs de cardiologie, Hôpital Louis Pradel, Bron, France; i Hospices Civils de Lyon, Anesthésie-Réanimation Neurologique, Hôpital Pierre Wertheimer, Bron, France; j Hospices Civils de Lyon, Anesthésie-Réanimation cardio-thoracique et vasculaire, Hôpital Louis Pradel, Bron, France; k Hospices Civils de Lyon, Anesthésie-Réanimation, Hôpital Edouard Herriot, Lyon, France; l Hospices Civils de Lyon, Anesthésie-Réanimation, Hôpital de la croix rousse, Lyon, France; m Hospices Civils de Lyon, Médecine Interne, Hôpital Edouard Herriot, Lyon, France.

**Keywords:** auto-immune disease flare-up, critical illness, cardiovascular diseases, comorbidities, long term prognosis, sepsis, systemic rheumatic diseases

## Abstract

Patients with systemic rheumatic disease (SRD) share the risks of multi-organ flare-up, cardiovascular diseases, and immunosuppression. Such situations can lead to an acute critical illness. The present study describes the clinical features of SRD patients admitted to the intensive care unit (ICU) and their short- and long- term mortality.

We performed a multicentre retrospective study in 10 French ICU in Lyon, France. Inclusion criteria were SRD diagnosis and admission for an acute organ failure. The primary endpoint was ICU mortality.

A total of 271 patients were included. SRD included systemic lupus erythematosus (23.2% of included patients), vasculitis (10.7%), systemic sclerosis (10.7%), idiopathic inflammatory myopathy (6.3%), and other connective tissue disorders (rheumatoid arthritis, Sjögren and Sharp syndromes; 50.9%). Initial organ failure(s) were shock (43.5% of included patients), acute kidney injury (30.5%), and acute respiratory failure (23.2%). The cause(s) of ICU admission included sepsis (61.6%), cardiovascular events (33.9%), SRD-flare up (32.8%), and decompensations related to comorbidities (28%). The ICU mortality reached 14.3%. The factors associated with ICU mortality were chronic cardiac failure, invasive ventilation and admission in ICU for another reason than sepsis or SRD flare-up. The median follow-up after ICU discharge was 33.6 months. During follow-up, 109 patients died. The factors associated with long-term mortality included age, Charlson comorbidity index, and ICU admission for sepsis or SRD flare-up.

The ICU mortality of patients with SRD was low. Sepsis was the first cause of admission. Cardiovascular events and comorbidities negatively impacted ICU mortality. Admission for sepsis or SRD flare-up exerted a negative effect on the long-term outcome.

## 1 Introduction

Systemic rheumatic diseases (SRD) are rare and heterogeneous diseases. They can affect multiple vital organs, depending on the nosological framework. Patients with SRD are significant healthcare consumers, since they represent 7% of the intensive care unit (ICU) admissions.^[1]^ All-cause ICU admissions concern 13,8% of hospitalizations in this population.^[2]^ Considering the occurrence of acute critical illness, SRD are inconsistently found a homogeneous group of chronic diseases.^[1,3,4]^ Studies remain controversial regarding whether, among ICU-admitted patients, SRD patients are intrinsically more severe than non-SRD patients.^[5,6]^ Nonetheless, SRD patients share the risks of severe acute multi-organ flare-up related to the underlying immune disorder, chronic organ failure, or severe immunosuppressive drugs side effects. Such situations may induce life-threatening conditions requiring ICU admission. SRD patients might display specificities regarding their healthcare requirements, as well as their short- and long-term prognosis: the intensivists concerns are to determine which characteristics are associated with ICU-mortality, and the internists need to identify which categories of patients will undergo an unfavorable trend after ICU discharge, in terms of long-term mortality, morbidity, and SRD evolution. The present study aims at describing

1. the clinical features of SRD patients presenting with acute organ failure(s) leading to an ICU admission,
2. their short-term outcome after ICU admission and its determinants, and
3. the long-term outcome of ICU survivors in terms of mortality and morbidity.

## 2 Methods

A retrospective multicentre observational study was conducted herein. Medical records of consecutive patients admitted to 10 medical university ICU departments within Hospices Civils de Lyon (Lyon, France) for a first ICU admission between 01/01/2011 and 31/12/2015 were reviewed. Patients were pre-selected from the *programme de médicalisation des systèmes d’information* (PMSI, a French medical information system program data).

### 2.1 Inclusion criteria

Patients were included in the study if they met all the following inclusion criteria: age ≥16 years, first admission in the ICU for acute organ failure(s) during the considered period (no duplicate), and diagnosis of SRD (diagnosed before or during the hospitalization). SRD included systemic lupus erythematosus (SLE), Sjögren syndrome (SS), systemic sclerosis (SSc), rheumatoid arthritis (RA), Sharp's syndrome, Anti-Neutrophil Cytoplasmic Antibodies -associated vasculitis, Goodpasture syndrome, non-type 1- and non-Hepatitis C Virus-related cryoglobulinemia, and inflammatory myopathies.^[7]^ Such diagnoses were made according to international criteria.^[8–12]^ Other SRD for which the pathophysiology does not involve a proven humoral auto-immunity or that are not susceptible to trigger multi-organ damage were not considered. Organ failure should be present at admission in the ICU, according to the Sequential Organ Failure Assessment (SOFA) score items^[13]^ (at least one item should be >1). Patients presenting peri-operative critical illness, were not included.

### 2.2 Data collection

Data and patient information were retrospectively collected in medical charts

1. upon ICU admission (demographic data, SRD characteristics, Charlson Comorbidity Index items,^[14]^ organ failure(s) requiring ICU admission, new Simplified Acute Physiology Score^[15]^ and SOFA score,^[13]^ and immunosuppressive treatments such as immunosuppressive drugs (methotrexate, azathioprine, cyclophosphamide, leflunomide, cyclosporine, tacrolimus, mycophenolate mofetil and mycophenolic acid), monoclonal antibodies (Tumor Necrosis Factor (TNF)α-blockers, Interleukin 6-blockers or B-cell depletion) and corticosteroid daily doses),
2. on the first 24 hours following ICU admission (initial diagnoses among sepsis (defined following Sepsis-3 international criteria^[16]^), cardiovascular events (acute coronary syndrome, acute cardiac rhythm disorder, stroke), decompensations associated to a comorbidity (chronic lung disease exacerbation, chronic heart failure decompensation, acute kidney injury during chronic kidney disease, and cirrhosis decompensation) and SRD flare-up – defined following clinicians conclusions),
3. during the ICU stay (organ supply techniques, occurrence of new organ failures (i.e., increase of ≥1 point of ≥1 SOFA item), nosocomial ICU-acquired sepsis (i.e., sepsis^[16]^ diagnosed after the first 48 hours of ICU stay), cardiovascular events, significant haemorrhagic complications (i.e., requiring transfusion), complications of invasive procedures (pneumothorax, gas embolism, punction site bleeding, catheter related infection) and drug side-effects),
4. about the vital status at ICU-discharge, and
5. regarding long-term outcome (survival after ICU discharge, occurrence of infectious events (i.e., local or systemic infection requiring non-programmed hospitalization or occurrence of infection during hospitalization), significant SRD flare-up (i.e., requiring an increase in immunosuppressive regimen and a non-programmed hospitalization), diagnosis of cardiovascular events, drugs-related side effects, cancer or hemopathy, and ICU readmission.

Follow-up was completed on 30/01/2018 by consulting the medical records. On 30/01/2018, a mortality request was addressed to the national registry of births and deaths, avoiding a loss of data regarding mortality.

### 2.3 Statistical analysis

The primary endpoint was ICU mortality. Secondary endpoints were survival after ICU discharge and failure rate after ICU discharge regarding SRD flare-up. Normal distribution was verified using the Kolmogorov-Smirnov test and graphically. Qualitative variables were compared using χ^2^-test or Fisher exact test. Quantitative variables were compared using Student *t* test or Wilcoxon non-parametric test. A univariable logistic regression was conducted. To identify the risk factors independently associated with the outcomes, clinically relevant variables that were significant according to a univariable analysis (threshold 0.2) were subjected to a multivariable backward stepwise logistic regression analysis. Long-term prognostic factors were assessed using a semi-parametric Cox model, after verification of risk-proportionality hypothesis. The Kaplan–Meier method was performed to obtain survival curves, that were compared using the log-rank test. Statistical tests were two-sided and the significance threshold was set to a *P* value ≤.05. Results are displayed with odds ratio (OR) or hazard ratio (HR) and their 95% confidence interval (95% CI). Statistical analyses were carried out using the STATA software (version 12, StataCorp, College Station, Texas).

### 2.4 Ethical considerations

Data collection was approved by the *Comission Nationale de l’Informatique et des Libertés* (CNIL HCL 16-085). Due to retrospective settings, the need for patient information and consent was waived, following the current French legislation.

## 3 Results

### 3.1 Description of the cohort

From 724 reviewed ICU records, 271 patients met the inclusion criteria (see Figure S1, Supplemental content which displays the flow chart of the study). Patients’ characteristics at baseline are described in Table 1. The mean age of the included participants was 64.1 ± 16.4 years and 34.3% of them were male. SRD were clustered into 5 groups: SLE with or without other auto-immune disorders (23.2%), vasculitis (including Anti-Neutrophilic Cytoplasm Antibodies-associated, cryoglobulinemia, and Goodpasture's vasculitis; 10.7%), SSc (8.9%), idiopathic inflammatory myopathies (dermatomyositis, polymyositis or anti-synthetase syndrome; 6.3%) and other connective tissue diseases (including RA, SS, Sharp; 50.9%). For 7.7% of cases, the diagnosis of SRD was made upon ICU admission. Patients were treated with corticosteroids alone in 22.1% of cases, a combination of corticosteroids and/or other immunosuppressive drugs (38.0% of cases), or monoclonal antibodies (11.8% of cases). Patients suffered from comorbidities: chronic pulmonary disease (43.5%), chronic cardiac failure (37.2%), chronic kidney disease (28.3%), diabetes (17.8%), cerebrovascular disease (17.5%), and/or coronary heart disease (17.1%).

**Table 1:** Baseline characteristics.

Characteristics	Value
General characteristics	
Age (yr)	64.6 ± 16,4
Sex (male)	93 (34.3%)
SRD characteristics	
Nature of the SRD:	
- Systemic lupus erythematosus	63 (23.2%)
- Vasculitis	29 (10.7%)
- Systemic sclerosis	24 (8.9%)
- Idiopathic inflammatory myopathy	17 (6.3%)
- Other connective tissue disorders	138 (50.9%)
Rheumatoid arthritis	112
Sjögren syndrome	17
Sharp syndrome	2
Main organ(s) affected by SRD:	
- Lungs^∗^	100 (36.9%)
- Kidneys^†^	78 (28.7%)
- Heart ^‡^	75 (27.6%)
- Nervous system^§^	50 (18.4%)
Duration of SRD before ICU admission (years)	11.7 ± 13.6
SRD diagnosed upon ICU admission	21 (7.7%)
Recently diagnosed SRD^||^	33 (12.2%)
Long-diagnosed SRD^¶^	217 (80.1%)
Immunosuppressive treatment:	
- corticosteroids alone	60 (22.1%)
- other immunosuppressive drugs	103 (38.0%)
- monoclonal antibody	31 (11.8%)
Comorbidities	
Charlson comorbidity index	4.5 ± 2.5
- Chronic pulmonary disease	118 (43.5%)
- Chronic cardiac failure	101 (37.2%)
- Chronic kidney disease	77 (28.4%)
- Diabetes	48 (17.7%)
- Cerebrovascular disease	47 (17.3%)
- Coronary heart disease	46 (17.0%)

Values are mean ± SD or counts (percentage).

∗ Current or history of diffuse interstitial pneumonia, pulmonary hypertension, shrinking lung syndrome, or pulmonary vasculitis.

† Current or history of glomerulitis, lupus nephropathy, interstitial nephritis, vascular nephropathy, or sclerodermic crisis.

‡ Current or history of pericarditis, myocarditis, endocarditis, or coronary vasculitis.

§ Current or history of cerebritis, myelitis, cerebral vasculitis, meningitis, meningoradiculitis, or peripheral nerve inflammation.

|| Diagnosed <2 months before ICU admission.

¶ Diagnosed ≥2 months before ICU admission. ICU = intensive care unit, SRD = systemic rheumatic disease.

### 3.2 Critical illness and ICU stay

The organ failure(s) leading to the ICU admission could be multiple and included shock (43.5%), acute kidney injury (30.3%), acute respiratory failure (23.2%), and neurological failure (20.7%). The cause(s) of admission into the ICU could be multiple and were attributed to sepsis (61.6%), SRD flare-up (32.8%; a combination of both causes were observed in 13.7% of cases), cardiovascular events (33.9%) and decompensations associated to comorbidities (28.0%). These causes were clustered into 3 groups based on clinical presentation: 89 patients with SRD flare-up (with or without other causes; 32.8%), 130 with Sepsis (without SRD manifestation, with or without other causes; 48.0%), and 52 with non-SRD and non-septic related causes (decompensation related to comorbidity and/or cardiovascular event, without sepsis and without SRD flare-up; 19.2%) (see Table S1, Supplemental content which describes the initial critical illness and characteristics of ICU stay, and Table S2, Supplemental content which displays the features of SRD flare-up, sepsis, and other reasons for ICU admissions). Mechanical invasive ventilation was required in 43.9% of cases and renal replacement therapy in 22.1%. During their ICU stay, patients presented with an ICU-acquired nosocomial sepsis in 25.8% of cases, a new cardiovascular event in 36.2% of cases, a significant bleeding in 25.5% of cases, a drug-related side effect in 13.6% of cases, and a complication of an invasive procedure in 11.8%. ICU-stay mean ± SD duration was 7.4 ± 11.8 days.

### 3.3 Short-term mortality

So as to answer to the intensivists concerns, we studied ICU mortality and its determinants. Thirty nine patients died during their stay in the ICU (14.3%). Univariable analysis was performed to evaluate the relationship between SRD characteristics, comorbidities, severity of initial critical illness, and causes for ICU admission, and ICU mortality (see Table S3, Supplemental content which compares ICU survivors and non-survivors using an univariable logistic regression). None of the SRD characteristic was associated with a significantly greater risk of ICU-mortality. Being admitted in ICU for a reason other than SRD flare-up or sepsis was associated with a greater risk of mortality (OR 2.56; 95% CI: [1.12 – 5.84]; *P* = .03). ICU survivors and non-survivors are compared in Table 2 using a multivariable logistic regression. Here again, none of the SDR characteristics was associated with a significantly greater risk of mortality. The SOFA score (OR 1.33; 95% CI: [1.18–1.51]; *P* = .02), history of cardiac failure (OR 2.56, 95%CI: [1.14–5.73]; *P* = .02), need for invasive mechanical ventilation (OR 6.15; 95% CI: [2.28–16.57]; *P* < .001), and non-septic non-SRD-flare-up critical illness (OR 3.34; 95% CI: [1.20–9.30]; *P* = .02) were associated with a greater risk of mortality.

**Table 2:** Multivariable analysis of mortality at ICU discharge.

Variable	Odds ratio	95% confidence interval	*P*
General			
Age (yr)	1.01	0.99–1.04	.45
Sex (male)	1.19	0.50–2.81	.69
*(reference: female)*			
Comorbidities			
Chronic heart failure	**2.56**	**1.14–5.73**	**.02**
*(reference: no chronic heart failure)*			
Cause of initial ICU admission			
SRD flare-up^∗^	1.53	0.54–4.32	.43
Non-septic and non SRD-associated critical illness^†^	**3.34**	**1.20–9.30**	**.02**
*(reference: sepsis (without SRD flare-up))*			
Severity of critical illness and ICU stay			
SOFA score	**1.33**	**1.18–1.51**	**.002**
Invasive mechanical ventilation	**6.15**	**2.28–16.57**	**.0003**
*(reference: no invasive ventilation)*			

R^2^ of the model: 0.80. Statistically significant comparisons are bold.

∗ With or without associated sepsis.

† That is, decompensations associated to comorbidities and cardiovascular events, without manifestations of SRD flare-up or sepsis. ICU = intensive care unit, SOFA = sequential organ failure assessment, SRD = systemic rheumatic disease.

### 3.4 Long term survival

**Figure 1:**
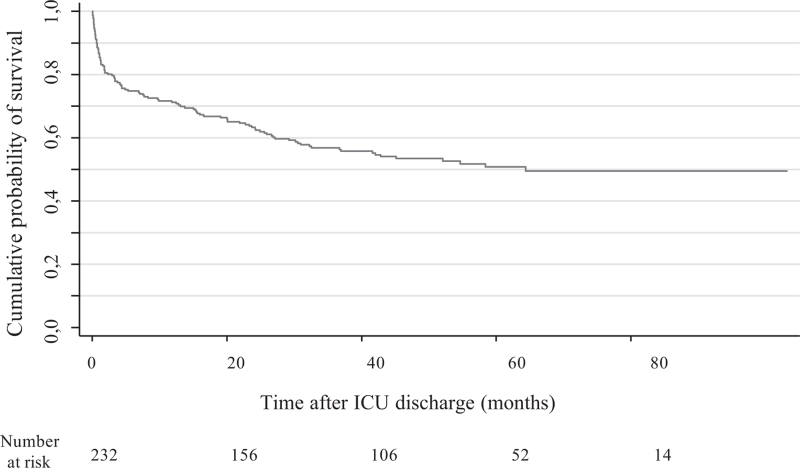
Kaplan–Meier graph of cumulative survival probability after ICU discharge in patients with systemic rheumatic diseases. ICU = intensive care unit.

So as to answer to the internists and rheumatologists concerns, who usually take care of SRD patients after an ICU stay, we described and analyzed the long term-mortality in ICU survivors. Survival data were available in the 232 patients that survived their ICU stay, and the median follow-up duration was 33.6 months from ICU discharge. During follow-up, 109 patients died, among them 26 patients died during the same hospitalization after ICU-discharge. One-year survival was 70.3%. The median survival was 64.4 months (interquartile range: [5.3–undetermined]). Figure 1 illustrates the survivor curve of participants after ICU discharge determined using the Kaplan–Meier method. Univariable analysis was performed to evaluate the relationship between patients characteristics, SRD characteristics, comorbidities, severity of initial critical illness and of ICU stay and long-term mortality of ICU survivors (see Table S4, Supplemental content which displays the results of univariable analysis using Cox's proportional hazards model to identify variables predictive of long-term mortality after ICU discharge). Table 3 shows the results of the multivariable analysis. None of the SDR characteristics was associated with a significantly greater risk of long-term mortality. Age, (HR 1.02; 95%CI: [1.01–1.04]; *P* = 004), the Charlson comorbidity index (HR 1.11; 95% CI: [1.04–1.19]; P=.004) exerted a negative effect, and admission in ICU for other reasons than sepsis and SRD flare-up were found to exert a protective effect regarding long-term mortality (HR 0.46; 95% CI: [0.25–0.85]; *P* = 01).

**Table 3:** Multivariable analysis of long-term survival after ICU discharge.

Variable	Hazard ratio	95% confidence interval	*P*
General			
Age (yr)	**1.02**	**1.01–1.04**	**.004**
Sex (male)	1.05	0.70–1.59	.80
*(reference: female)*			
Comorbidities			
Charlson comorbidity index	**1.11**	**1.04–1.19**	**.004**
Nature of SRD			
SLE	0.68	0.38–1.21	.19
*(reference: non-SLE SRD)*			
Cause of initial ICU admission			
SRD flare-up^∗^	0.99	0.64–1.53	.96
Non-septic and non SRD-associated causes^†^	**0.46**	**0.25–0.85**	**.01**
*(reference: sepsis (without SRD flare-up))*			

Statistically significant comparisons are bold.

∗ With or without sepsis associated.

† That is, decompensations associated to comorbidities and cardiovascular events, without manifestations of SRD flare-up or sepsis. ICU = intensive care unit, SOFA = sequential organ failure assessment, SRD = systemic rheumatic disease.

### 3.5 SRD flare-up incidence after ICU discharge

**Figure 2:**
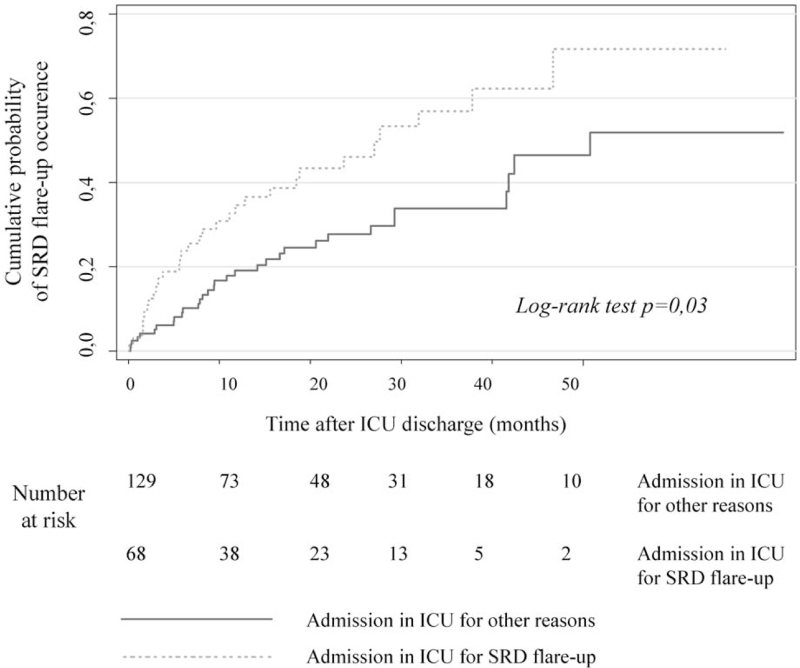
Kaplan–Meier cumulative failure curves for SRD flare-up after ICU discharge in patients with SRD admitted in ICU for a critical illness attributed to a SRD flare-up (light grey dash line) and to other causes but SRD flare-up (dark grey continuous line). ICU = intensive care unit, SRD = systemic rheumatic diseases.

An ICU stay is often a circumstance of inflammatory triggers of SRD flare-up, stress-induced immunosuppression, and modifications of the immunosuppressive therapy: the internists that are in charge of ICU survivors with SRD need to identify which subgroup is at highest risk for SRD flare-up. We herein described and analyzed the SRD flare-up incidence during follow up. Clinical data during follow-up after ICU discharge were available for 197 patients. Median SRD flare-up free progression was 41.2 months. Figure 2 illustrates the cumulative failure curves determined using the Kaplan–Meier method regarding the occurrence of SRD flare-up after ICU discharge. Patients admitted in ICU for SRD flare-up presented a significantly higher risk for SRD flare-up after ICU discharge than patients admitted in ICU for other reasons (*P* = .03). SRD flare-up as the initial cause for ICU admission (HR 1.97; 95% CI: [1.06–3.66]; *P* = .03), occurrence of sepsis during ICU-stay (HR 2.31; 95% CI: [1.29–4.13]; *P* = .005), and treatment with non-steroid immunosuppressive drugs at ICU admission (HR 2.17; 95% CI: [1.21–3.89]; *P* = .009) were independently associated with the occurrence of SRD flare-up after ICU discharge. Recently diagnosed SRD were not significantly associated with the occurrence of SRD flare-up compared to long-diagnosed SRD (See Tables S5 and S6, Supplemental content which display the results of univariable and multivariable analysis respectively concerning cumulative probability of SRD flare-up during follow-up after ICU discharge).

### 3.6 Morbidity 12 months after ICU discharge

SRD it-self might be a threat to these patients, after ICU discharge, and so might be comorbidities, septic events, cardiovascular events, and immunosuppression burden. We herein describe morbidity at a point 12 months after ICU discharge. Clinical data 12 months after ICU discharge were available for 133 patients. One year after ICU discharge, 24.9% of at-risk patients had presented an SRD flare-up, the median corticosteroid daily dose at 1 year was 5 mg (interquartile range: [0–10]), and 44.4% of patients underwent a non-steroid immunosuppressive treatment. One year after ICU discharge, 46.8% of at-risk patients had presented with an infectious event, 24.1% a severe drug side-effect, 7.5% a cardiovascular event, 28.2% were re-admitted in the ICU, and 17% were diagnosed with a solid malignant tumor or hemopathy.

## 4 Discussion

In the present study, the clinical features of SRD patients presenting with acute organ failure(s) leading to an ICU admission were predominantly attributed to sepsis, followed by SRD flare-up and decompensations associated to comorbidities. Their mortality in ICU was low, and was negatively impacted by cardiovascular events and comorbidities. Admission in the ICU for sepsis or SRD flare-up exerted a negative effect on the long-term outcome. We have highlighted new findings concerning the contribution of comorbidities and cardiovascular risk in ICU mortality, as previously reported in general settings.^[25–28]^ We also outlined the contributors to long-term mortality and morbidity, after ICU discharge.

### 4.1 Causes of ICU admission

The observations made in the present study regarding the causes for ICU admission are partially consistent with the literature:^[1,17–24]^ sepsis is regularly reported as the first cause of ICU admission in these patients, followed by SRD flare-up. Almost half of the patients presenting SRD flare-up at ICU-admission were also suffering from sepsis, highlighting difficulties for clinicians to distinguish one from the other. Septic patients also presented with cardiovascular events and decompensations associated to comorbidities, which represented a larger proportion of the causes of admission compared to what is usually reported in the literature.

### 4.2 Short- and long-term survival

The present study analyzes one of the largest European cohorts of critically ill SRD patients.^[17,18,24]^ A huge improvement has been made in critically ill SRD patients care in the last few years. Thirty years ago, Godeau et al reported an ICU mortality of 33%^[29]^ while recent studies account for a much lower mortality, around 20%.^[17,18]^ We herein report a particularly low in-ICU mortality (14.3%), which can be due to the recruitment criteria of our cohort: a large proportion of patients presented RA or SS, which rarely induce critical end-organ damages. However, this observation should be put into perspective with the noticeably higher in-hospital mortality (24%): the patients who are discharged alive from the ICU still present a high risk of mortality shortly after their transfer in conventional wards, as previously observed^[19,20]^ illustrating the special care that should be taken by intensivists before discharging them in conventional wards. Our one-year survival result of 70.3% was comparable to previously published data that showed an excellent long-term outcome for ICU survivors.^[17,24]^

### 4.3 Prognostic contribution of SRD characteristics on short-term mortality

The contribution of individual SRD characteristics to short-term mortality remains debated in the literature: while some authors reported that, in the ICU, mortality was very rarely attributed to the SRD itself,^[21,30]^ others have associated SRD flare-up in ICU with mortality,^[18]^ or found that 30% of deaths were ascribable to SRD exacerbation.^[17]^ Heijnen et al^[19]^ reported that the highest hospital mortality concerned SSc patients, and the lowest patients with SLE. Contradictorily, Faguer et al^[18]^ found a significant association between dermatomyositis and 30-days mortality and no association for other SRD. In the present study the highest mortality was found for patients suffering from SSc, followed by idiopathic inflammatory myositis, and the lowest for patients suffering from SLE and RA, SS, and Sharp, but these rates were not significantly different, and multivariable analyses disproved an independent association between the nature of the SRD and ICU mortality. These data herein suggest that the impact of SRD characteristics on short-term prognosis is, at best, limited.

### 4.4 Prognostic contributions of comorbidities and cardiovascular diseases

Patients admitted in the ICU for reasons other than sepsis or SRD flare-up displayed the poorest short-term prognosis. History of myocardial infarction or chronic cardiac failure, and cardiovascular events as cause for ICU admission or cause of complications during ICU stay were significantly associated with ICU mortality. The cardiovascular risk in this specific population should therefore be taken into account by intensivists with particular attention, as it appears to strongly influence the ICU outcome.

Although SRD patients often present with comorbidities, few studies have investigated the prognostic contribution of comorbidities in these patients.^[22,29,31]^ The presence of a previous chronic comorbidity has been believed to be associated with vital prognosis^[32]^ but there is no published study that allows to distinguish a contribution on short-term or long-term mortality specifically. The present study suggests that non-cardiac comorbidities are not associated with an increased risk of in-ICU mortality, unless they are the cause of the initial critical illness. Nonetheless, they and cardiovascular comorbidities are associated with a long-term prognostic impairment.

### 4.5 Long-lasting risk for SRD exacerbation, and morbidity after ICU discharge

For ICU survivors, suffering from a severe SRD flare-up leading to a critical illness were associated with a greater risk of mortality in the long-term, and SRD flare-up as a cause of initial ICU admission was associated with a poorer SRD flare-up-free survival, as it was the case for immunosuppressive treatments. This suggests that a severe SRD flare up leading to ICU admission is hardly controlled on the long-term despite a high immunosuppressive burden, which is in accordance with similar factors and conclusions from another study.^[33]^ Moreover, patients suffering from sepsis during their ICU stay showed a higher rate of SRD flare-up during follow-up. Internists should take great care to these patients as well: the presumed septic risk in these highly frail patients might prevent them from taking full advantage of an optimal immunosuppressive therapy.

The present study highlights a high incidence of septic, cardiovascular, oncologic complications 1 year after ICU discharge. Also, patients with SRD were subjected to a high iatrogenic risk both during the ICU stay and after ICU discharge. The improvements that have been made in treatments for SRD exacerbation- or sepsis-induced acute critical illness permit a majority of patients to be discharged alive, but such situations are indicators of frailty in SRD patients, which manifests as a poorer long-term prognosis. The latter can be explained by the SRD itself, premature cardiovascular diseases and comorbidities, as well as by the treatments.

### 4.6 Limitations and strengths

The present study may suffer some methodological weaknesses. Firstly, the retrospective design may have altered the quality of data collection and results and prevented from collecting useful data such as the quality of life of ICU survivors. Nevertheless, a prospective scheme would not have allowed such an exhaustive collection for such rare diseases. Furthermore, data collection was systematic and made by 1 single investigator, ensuring reduction of information bias. Secondly, the ICU wards that contributed to this study were all university ICU, which potentially hinders generalization of our results to other settings. Nevertheless, these ICU wards account for most of ICU services offered in the geographic area. Such geographic characteristics facilitated the acquisition of long-term follow-up data, and diminished the risk of loss for follow-up. Finally, the high number of comparisons might have induced some artificial positive results.

## 5 Conclusion

ICU mortality of SRD patients was low, and their long-term survival was good, justifying their admission in the ICU. However, care should be taken while discharging them from the ICU to conventional wards, as the in-hospital mortality was relatively high.

Comorbidities and cardiovascular diseases played a significant negative role, predominantly on the short-term outcome, when they were responsible for the initial acute critical illness. On the contrary, SRD flare-up and sepsis, when responsible for the initial critical illness, exerted a long-term negative effect on mortality and morbidity that has to be taken into account by the internists that will take care of them after ICU discharge.

Prospective studies on SRD patients admitted in ICU would be helpful to confirm our results and to clarify the differential prognostic contributions of their clinical characteristics on the long term.

## Acknowledgments

We thank Hélène BOYER (DRCI, Hospices Civils de Lyon) for help in manuscript preparation.

## Author contributions

**Conceptualization:** Paul Chabert, Arnaud Hot.

**Data curation:** Paul Chabert, William Danjou, Audrey Bestion, Abla-Akpene Fred.

**Formal analysis:** Paul Chabert, Julien Berthiller.

**Investigation:** Paul Chabert.

**Methodology:** Paul Chabert, Mehdi Mezidi, Julien Berthiller, Audrey Bestion, Abla-Akpene Fred, Arnaud Hot.

**Project administration:** Paul Chabert.

**Resources:** Paul Chabert, Claude Guerin, Laurent Argaud, Vincent Piriou, Eric Bonnefoy-Cudraz, Jean-Jacques Lehot, Jean-Luc Fellahi, Thomas Rimmelé, Frederic Aubrun, Jean-Christophe Richard.

**Supervision:** Laure Gallay, Arnaud Hot.

**Validation:** Arnaud Hot.

**Visualization:** Arnaud Hot.

**Writing – original draft:** Paul Chabert.

**Writing – review & editing:** Paul Chabert, Mehdi Mezidi, Laure Gallay, Arnaud Hot.

## Supplementary Material

Supplemental Digital Content

## Supplementary Material

Supplemental Digital Content

## Supplementary Material

Supplemental Digital Content

## Supplementary Material

Supplemental Digital Content

## Supplementary Material

Supplemental Digital Content

## Supplementary Material

Supplemental Digital Content

## Supplementary Material

Supplemental Digital Content
