# Supplementary figures and images for: Short- and long-term prognosis of acute critically ill patients with systemic rheumatic diseases: A retrospective multicentre study
Source: Medicine (Baltimore). 2021 Sep 3;100(35):e26164. doi: 10.1097/MD.0000000000026164 (PMC8415942; doi:10.1097/MD.0000000000026164)

**FIGURE 2. FLOW CHART**

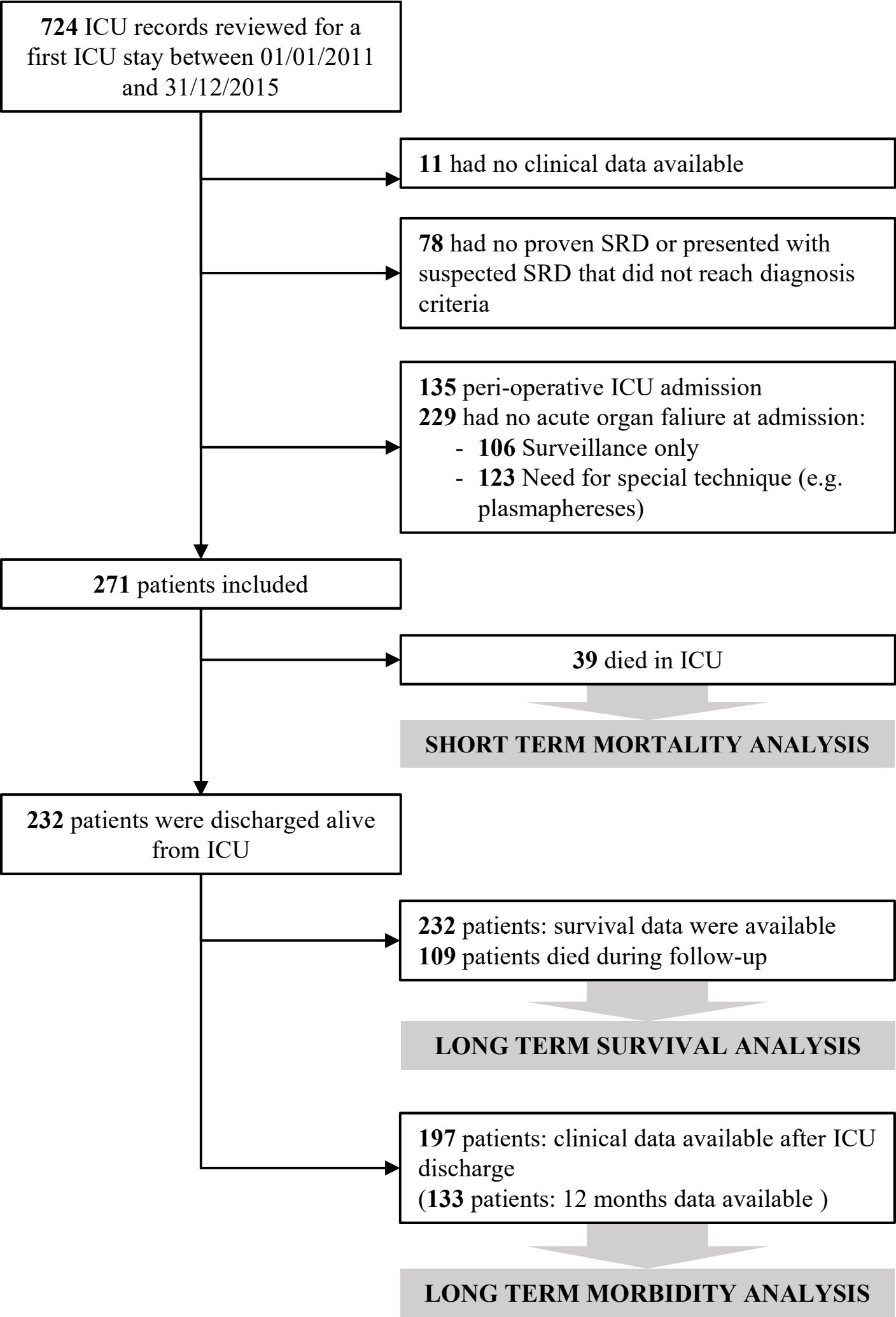

Supplement: Supplemental Digital Content [file medi-100-e26164-s001.pdf]
