# Supplementary material for: Short- and long-term prognosis of acute critically ill patients with systemic rheumatic diseases: A retrospective multicentre study
Source: Medicine (Baltimore). 2021 Sep 3;100(35):e26164. doi: 10.1097/MD.0000000000026164 (PMC8415942; doi:10.1097/MD.0000000000026164)
Supplement: Supplemental Digital Content [file medi-100-e26164-s002.pdf]

**Supplemental content - Table 1.** Description of initial critical illness and characteristics of ICU stay

| CHARACTERISTICS | Value |
|---|---|
| Critical illness at ICU admission | |
| SOFA Score [a] | 6.1 ± 3.1 |
| SAPS II score [b] | 43,8 ± 21,2 |
| Organ failures at admission: | |
| - Shock [c] | 118 (43.5%) |
| - Acute Kidney Injury [d] | 82 (30.3 %) |
| - Acute Respiratory failure [e] | 63 (23.2 %) |
| - Hemostasis disturbances [f] | 63 (23.2 %) |
| - Acute Neurological failure [g] | 56 (20.7 %) |
| - Liver Failure [h] | 16 (5.9%) |
| Cause(s) of ICU admission: | |
| - SRD Flare-up | 89 (32.8 %) |
| SRD Flare-up without sepsis | 52 |
| SRD Flare-up with sepsis | 37 |
| - Sepsis | 167 (61.6%) |
| - Cardiovascular event | 92 (33.9 %) |
| - Decompensations associated to comorbidities | 76 (28.0 %) |
| These causes were clustered in: | |
| - SRD flare-up (with or without other causes) | 89 (32.8%) |
| - Sepsis (without SRD flare-up, with or without other causes) | 130 (48.0%) |
| - Other causes (decompensation related to comorbidity and/or cardiovascular event only) | 52 (19.2%) |
| ICU stay duration (days) | 7.4 ± 11.8 |
| Notable events during ICU stay: | |
| - Nosocomial ICU-acquired sepsis [i] | 70 (25.8%) |
| - Occurrence of new organ failure(s) [j] | 125 (46.1%) |
| Invasive mechanical ventilation support | 119 (43.9%) |
| Renal replacement therapy | 60 (22.1%) |

Values are mean ± SD or count (percentage)

[a] At ICU admission

[b] During the first 24 hours of ICU stay

[c] Hypotension requiring vasoactive drugs

[d] Creatininemia > 170 µmol/L or urine output < 500mL/24h

[e] $PaO_2$ (mmHg)/$FiO_2$ < 300 or assisted ventilation

[f] Platelets < 100 G/L

[g] Glasgow conscience score < 13

[h] Total bilirubinemia > 33 µmol/L

[i] Occurring after the first 48 hours after ICU admission

[j] Occurring after the first 24 hours after ICU admission

Abbreviations: $FiO_2$: oxygen fraction in inspired air; ICU: intensive care unit; $PaO_2$: oxygen arterial pressure; SAPS II: simplified acute physiology score II; SOFA: sequential organ failure assessment; SRD: systemic rheumatic disease
