# Supplementary material for: Short- and long-term prognosis of acute critically ill patients with systemic rheumatic diseases: A retrospective multicentre study
Source: Medicine (Baltimore). 2021 Sep 3;100(35):e26164. doi: 10.1097/MD.0000000000026164 (PMC8415942; doi:10.1097/MD.0000000000026164)
Supplement: Supplemental Digital Content [file medi-100-e26164-s003.pdf]

Supplemental content - Table 2. Clinical features at ICU admission

CLINICAL FEATURE[a]	Number of cases
SRD Flare-up responsible for ICU admission (with or without sepsis)	
Acute intersticial pneumonia	14
Alveolar haemorrage	10
Vasculitis with pneumo-renal syndrome	10
Cerebritis/cerebral vasculitis	9
Glomerulonephritis	8
Catastrophic anti-phospholipid syndrome	5
Severe haemolysis	5
Tamponade	5
Myocarditis	5
Lympho-histiocytic activation syndrome	3
Auto-immune hepatitis	3
Bowel vasculitis with perforation	3
Pulmonary hypertension	3
Renal sclerodermic crisis	3
Interstitial nephritis	3
Sepsis responsible for ICU admission (without SRD manifestation)	
Bacterial/viral pneumonia	34
Bowel infection/peritonitis	19
Urosepsis	18
Bacteriemia of unknown origin	14
Catheter related infection	13
Angiocholitis/cholecystitis	9
Arthritis with bacteriemia	7
Necrotizing dermohypodermitis	5
Infectious encephalitis	5
Pneumocystosis	4
Listeriosis	1
Malaria	1
Non-septic non-SRD-related causes for ICU admission	
Cardiac decompensation	14
Chronic respiratory disease decompensation	13
Stroke	7
Chronic renal disease decompensation and/or severe hydro-electrolytic disorders	6
Cirrhosis decompensation	4
Non intentional drug overdosis	4
Severe drug reaction	2
Acute pancreatitis	1

Abbreviations: ICU: intensive care unit; SRD: systemic rheumatic disease.

[a] Main diagnosis; could be associated with other causes.
