# Supplementary material for: Short- and long-term prognosis of acute critically ill patients with systemic rheumatic diseases: A retrospective multicentre study
Source: Medicine (Baltimore). 2021 Sep 3;100(35):e26164. doi: 10.1097/MD.0000000000026164 (PMC8415942; doi:10.1097/MD.0000000000026164)
Supplement: Supplemental Digital Content [file medi-100-e26164-s004.pdf]

Supplemental content - Table 3. Univariable analysis of mortality upon ICU discharge

<u>VARIABLE</u>	**Odds Ratio**	**95% Confidence Interval**	**p**
<u>General</u>			
Age (years)	1.02	0.99-1.04	0.18
Sex (male)	1.08	0.53-2.20	0.82
(reference: female)			
<u>SRD Characteristics</u>			
SLE	0.69	0.29-1.64	0.40
SSc	1.65	0.58-7.71	0.35
Vasculitis	0.95	0.31-2.89	0.92
Inflammatory Myopathies	1.30	0.35-4.74	0.69
(reference: other connective tissue diseases (RA, SS, Sharp))			
Recently diagnosed SRD and SRD diagnosed in ICU [a]	0.17	0.02-1.24	0.08
(reference: long-diagnosed SRD [b])			
<u>Comorbidities</u>			
Charlson comorbidity index	1.07	0.94-1.22	0.33
History of myocardial infarction	**2.54**	**1.26-5.14**	**0.009**
(reference: absence of history of myocardial infarction)			
Chronic lung disease	1.12	0.56-2.25	0.75
(reference: absence of chronic lung disease)			
Chronic heart failure	**2.38**	**1.08-5.25**	**0.03**
(*reference: absence of chronic heart failure)*			
Chronic kidney disease	1.37	0.58-3.21	0.47
(reference: absence of chronic kidney disease)			
<u>Organ Failures at ICU admission</u>			
SAPS-II [c]	**1.05**	**1.03-1.07**	**<0.0001**
SOFA score [d]	**1.29**	**1.15-1.44**	**<0.0001**
Shock [e]	**4.66**	**2.17-10.02**	**<0.0001**
(reference: absence of shock)			
Acute respiratory failure [f]	1.19	0.55-2.61	0.66
(reference: absence of acute respiratory failure)			
Acute neurological failure [g]	**3.11**	**1.42-6.81**	**0.005**
(reference: absence of neurological failure)			
Acute kidney injury [h]	1.01	0.42-2.44	0.98
(reference: absence of acute kidney injury)			
<u>Cause of ICU admission</u>			
SRD flare-up	1.08	0.47-2.48	0.85
Non SRD-related and non-septic acute critical illness	**2.56**	**1.12-5.84**	**0.03**
(reference: sepsis without sign of SRD flare-up)			

Immunosuppressive treatment at ICU admission			
Steroid dose ≥ 20mg [i]	0.52	0.21-1.31	0.16
(reference: absence of steroid or dose < 20mg)			
Treatment with non-steroid immunosuppressive drugs [j]	1.25	0.64-2.47	0.51
(reference: absence of non-steroid drug)			
ICU stay			
New organ failures after the first 24 hours	**19.28**	**5.77-64.47**	**<0.0001**
(reference: absence of new organ failure)			
Renal replacement therapy	**2.26**	**1.09-4.70**	**0.03**
(reference: absence of renal replacement therapy)			
Invasive Mechanical ventilation	**9.27**	**3.73-23.03**	**<0.0001**
(reference: absence of invasive ventilation)			
Nosocomial ICU-acquired sepsis [k]	1.53	0.74-3.18	0.25
(reference: absence of nosocomial sepsis)			
Cardiovascular event occurring during ICU stay [l]	1.63	0.82-3.23	0.16
(reference: absence of cardiovascular event)			

[a] < 2 months before ICU admission

[b] ≥ 2 months before ICU admission

[c] Occurring during the first 24 hours after ICU admission

[d] At ICU admission

[e] Hypotension requiring vasoactive drugs (epinephrine, norepinephrine)

[f] PaO_2 (mmHg)/FiO_2 < 300 or assisted ventilation

[g] Creatininemia > 170 µmol/L or urine output < 500mL/24h

[h] Glasgow conscience score < 13

[i] Equivalent prednisone daily

[j] Among methotrexate, azathioprine, cyclophosphamide, leflunomide, cyclosporine, tacrolimus, mycophenolate mofetil, mycofenolic acid, Tumor Necrosis Factor α-blockers, Interleukin 6-blockers, or B-cell depletion

[k] Occuring after the first 48h after ICU admission

[l] Acute coronary syndrome, acute cardiac rhythm disorder, or stroke

Statistically significant comparisons are bold

Abbreviations: ICU: intensive care unit; RA: rheumatoid arthritis; SAPS II: simplified acute physiology score II; SOFA: sequential organ failure assessment; SLE: systemic lupus erythematosus; SRD: systemic rheumatic disease; SS: Sjögren Syndrome; SSc: systemic sclerosis
