# Supplementary material for: Short- and long-term prognosis of acute critically ill patients with systemic rheumatic diseases: A retrospective multicentre study
Source: Medicine (Baltimore). 2021 Sep 3;100(35):e26164. doi: 10.1097/MD.0000000000026164 (PMC8415942; doi:10.1097/MD.0000000000026164)
Supplement: Supplemental Digital Content [file medi-100-e26164-s005.pdf]

Supplemental content - Table 4. Univariable analysis of long-term survival after ICU discharge

VARIABLE	Hazard Ratio	95% Confidence Interval	p
General			
Age (years)	**1.02**	**1.01-1.04**	**<0.001**
Sex (male)	1.42	0.96-2.10	0.08
(reference: female)			
SRD Characteristics			
SLE	**0.45**	**0.26-0.78**	**0.05**
SSc	1.12	0.58-2.20	0.73
Vasculitis	0.81	0.44-1.51	0.51
Inflammatory Myopathies	1.69	0.84-3.41	0.14
(reference: other connective tissue diseases (RA, SS, Sharp))			
Recently diagnosed SRD and SRD diagnosed in ICU [a]	0.78	0.44-1.40	0.40
(reference: long-diagnosed SRD [b])			
Comorbidities			
Charlson comorbidity index	**1.14**	**1.06-1.22**	**<0.001**
History of myocardial infarction	1.36	0.84-2.22	0.21
(reference: absence of history of myocardial infarction)			
Chronic lung disease	**1.54**	**1.05-2.25**	**0.025**
(reference: absence of chronic lung disease)			
Chronic heart failure	**1.84**	**1.25-2.72**	**0.002**
(reference: absence of chronic heart failure)			
Chronic kidney disease	0.92	0.60-1.43	0.74
(reference: absence of chronic kidney disease)			
Organ Failures at ICU admission			
SAPS-II [c]	**1.01**	**1.00-1.02**	**0.02**
SOFA score [d]	1.06	0.99-1.13	0.08
Shock [e]	1.11	0.97-1.28	0.11
(reference: absence of shock)			
Acute respiratory failure [f]	1.06	0.90-1.23	0.48
(reference: absence of acute respiratory failure)			
Acute neurological failure [g]	1.12	0.95-1.31	0.17
(reference: absence of neurological failure)			
Acute kidney injury [h]	1.04	0.92-1.19	0.50
(reference: absence of acute kidney injury)			
Cause of ICU admission			
SRD flare-up	0.60	0.36-1.01	0.053
Non SRD-related and non-septic acute critical illness	**0.40**	**0.21-0.76**	**0.005**

(reference: sepsis without sign of SRD flare-up)			
<u>Immunosuppressive treatment at ICU admission</u>			
Steroid dose > 20mg [i]	1.25	0.82-1.91	0.28
(reference: absence of steroid or dose < 20mg)			
Treatment with non-steroid immunosuppressive drugs [j]	0.90	0.61-1.36	0.6
(reference: absence of non-steroid drug)			
<u>ICU stay</u>			
New organ failures after the first 24 hours	1.12	0.76-1.67	0.55
(reference: absence of new organ failure)			
Renal replacement therapy	1.50	0.96-2.35	0.076
(reference: absence of renal replacement therapy)			
Invasive Mechanical ventilation	1.34	0.91-1.98	0.135
(reference: absence of invasive ventilation)			
Nosocomial ICU-acquired sepsis [k]	**1.87**	**1.25-2.83**	**0.002**
(reference: absence of nosocomial sepsis)			
Cardiovascular event occurring during ICU stay [l]	1.22	0.82-1.82	0.32
(reference: absence of cardiovascular event)			
ICU stay duration (days)	0.99	0.98-1.01	0.83

Statistically significant comparisons are bold

[a] < 2 months before admission to the ICU

[b] ≥ 2 months before admission to the ICU

[c] Occurring upon the first 24 hours after ICU admission

[d] At ICU admission

[e] Hypotension requiring vasoactive drugs

[f] PaO2 (mmHg)/FiO_2 < 300 or assisted ventilation

[g] Creatininemia > 170 µmol/L or urine output < 500mL/24h

[h] Glasgow conscience score < 13

[i] Equivalent prednisone daily

[j] Among methotrexate, azathioprine, cyclophosphamide, leflunomide, cyclosporine, tacrolimus, mycophenolate mofetil, mycophenolic acid, Tumor Necrosis Factor α-blockers, Interleukin 6-blockers, or B-cell depletion

[k] Occuring after the 48 hours after ICU admission

[l] Acute coronary syndrome, acute cardiac rhythm disorder, or stroke

Abbreviations: ICU: intensive care unit; RA: rheumatoid arthritis; SAPS II: simplified acute physiology score II; SOFA: sequential organ failure assessment; SLE: systemic lupus

erythematosus; SRD: systemic rheumatic disease; SS: Sjögren Syndrome; SSc: systemic sclerosis
