# Supplementary material for: Short- and long-term prognosis of acute critically ill patients with systemic rheumatic diseases: A retrospective multicentre study
Source: Medicine (Baltimore). 2021 Sep 3;100(35):e26164. doi: 10.1097/MD.0000000000026164 (PMC8415942; doi:10.1097/MD.0000000000026164)
Supplement: Supplemental Digital Content [file medi-100-e26164-s006.pdf]

Supplemental content - Table 5. Univariable analysis of cumulative probability of SRD flare-up after ICU discharge

VARIABLE	Hazard Ratio	95% Confidence Interval	p
General			
Age (years)	0.99	0.97-1.01	0.37
Sex (male)	1.54	0.98-2.72	0.06
(reference: female)			
Nature of SRD Characteristics			
SLE	0.60	0.33-1.10	0.10
SSc	1.64	0.78-3.45	0.19
Vasculitis	1.24	0.61-2.52	0.61
Inflammatory Myopathies	1.22	0.44-3.37	0.69
(reference: other connective tissue diseases (RA, SS, Sharp))			
Recently diagnosed SRD and SRD diagnosed in ICU [a]	1.69	0.91-3.13	0.09
(reference: long-diagnosed SRD [b])			
Cause of ICU admission			
SRD flare-up	**1.97**	**1.20-3.24**	**0.007**
Non SRD-related acute critical illness	**0.40**	**0.21-0.76**	**0.005**
(reference: sepsis without sign of SRD flare-up)			
Immunosuppressive treatment at ICU admission			
Steroid dose > 20mg [c]	1.08	0.61-1.91	0.27
(reference: absence of steroid or dose < 20mg)			
Treatment with non-steroid immunosuppressive drugs [d]	1.27	0.77-2.10	0.34
(reference: absence of non-steroid drug)			
Characteristics of ICU stay			
Occurrence of sepsis during the ICU stay	**2.02**	**1.14-3.55**	**0.015**
(reference: no sepsis during the ICU stay)			
Steroid daily dose at ICU discharge	**1.01**	**1.00-1.02**	**0.02**
Decrease in immunosuppressive regimen [e]	1.00	0.49-2.05	0.98
Increase in immunosuppressive regimen [e]	**2.17**	**1.32-3.58**	**0.002**
(reference: no modification in the immunosuppressive treatment)			

Statistically significant comparisons are bold

[a] < 2 months before admission to the ICU

[b] ≥ 2 months before admission to the ICU

[c] Equivalent prednisone daily

[d] Among methotrexate, azathioprine, cyclophosphamide, leflunomide, cyclosporine, tacrolimus, mycophenolate mofetil, mycophenolic acid, Tumor Necrosis Factor α-blockers, Interleukin 6-blockers, or B-cell depletion

[e] Comparing the immunosuppressive therapy between the treatment at ICU admission and one week after ICU discharge (addition or suppression of one or more drugs, increase or decrease of the corticosteroid daily dose)

Abbreviations: ICU: intensive care unit; RA: rheumatoid arthritis; SLE: systemic lupus erythematosus; SRD: systemic rheumatic disease; SS: Sjögren Syndrome; SSc: systemic sclerosis
