# Supplementary material for: Short- and long-term prognosis of acute critically ill patients with systemic rheumatic diseases: A retrospective multicentre study
Source: Medicine (Baltimore). 2021 Sep 3;100(35):e26164. doi: 10.1097/MD.0000000000026164 (PMC8415942; doi:10.1097/MD.0000000000026164)
Supplement: Supplemental Digital Content [file medi-100-e26164-s007.pdf]

Supplemental content - Table 6. Multivariable analysis of cumulative probability of SRD flare-up after ICU discharge

**VARIABLE**	**Hazard Ratio**	**95% Confidence Interval**	**p**
General			
Age (years)	0.99	0.97-1.01	0.15
Sex (male)	0.70	0.41-1.19	0.18
(reference: female)			
Nature of SRD			
SLE	0.60	0.29-1.22	0.16
(reference: non-SLE SRD)			
Characteristics of SRD			
Treatment with non-steroid immunosuppressive drugs [a]	**2.17**	**1.21-3.89**	**0.009**
(reference: absence of non-steroid drug)			
Recently diagnosed [b] SRD or SRD diagnosed in ICU	1.56	0.70-3.47	0.277
(*reference: long-diagnosed [c] SRD)*			
Characteristics of ICU stay			
SRD flare-up cause of initial ICU admission	**1.97**	**1.06-3.66**	**0.03**
(reference: other causes of ICU admission)			
Occurrence of sepsis during the ICU stay	**2.31**	**1.29-4.13**	**0.005**
(reference: no sepsis during the ICU stay)			

[a] Among methotrexate, azathioprine, cyclophosphamide, leflunomide, cyclosporine, tacrolimus, mycophenolate mofetil, mycofenolic acid, Tumor Necrosis Factor α-blockers, Interleukin 6-blockers, or B-cell depletion

[b] < 2 months before ICU admission

[c] ≥ 2 months before ICU admission

Statistically significant comparisons are bold

Abbreviations: ICU: intensive care unit; RA: rheumatoid arthritis; SLE: systemic lupus erythematosus; SRD: systemic rheumatic disease; SSc: systemic sclerosis
